# On Vaccævaration

**Published:** 1804-12-01

**Authors:** 


### *To the Editors of the Medical and Physical Journal.*

Gentlemen,

THE Cow-pock having given rise to two parties, one of its prophylacticity, the other of its indifference, (which latter party, for the sake of humanity, it is hoped is fast sinking into greater indifference) three partisans also of a classical term for the disorder having arisen, be pleased once more to allow me to intervene between Dr. Walker and Mr. Ring; or, as the former might not disapprove of my expressing myself, to step into the ring, in behalf of VACCÆVARA.

Vara is *not* "a more precise term than the diminutive variola for the small-pox, (see Dr. W. Medical and Physical Journal, No. 69, p. 440) but it is a more precise term for the cow-pock, because the word variolæ respects the number of small pustules by which the skin is varied in the former, whilst VARA, not a diminutive, may better express the single and large eruption that appears in the latter. Vara *has* taken place in medical language, if the hand-writing of a medical man upon a paper of vaccævarous matter, *Vara vaccina*, which I have seen, is any authority. Vara is also as old as Pliny; but variol*a* is still newer than variol*æ*.

Vaccævāra may by use become as euphonous as venæsectio, to which, as a compound, it is analogous. Assĕveration, pronounced by quantity, would be nearly as cacophonous as vaccævāration; but, would any one therefore say, *assiolation*, and because of the similitude of this new coin to an established one? Thus *acceleration* too might be *acciolation*.

Classical minds will respect Dr. W. as a man of abilities; but, classical ears will not be *heifer'd*, if they can help it, with *vacciola*; any more than classical tastes will admit *vaccina* to express a disorder, and thereby to create a disgust against milk, butter, and cheese, those agreeables, of which alone, from the term, they had been accustomed to conceive ideas. But, vacc*ina*, even for the disorder, is much better than vacciola, the former being an established word, while the latter never can be any thing better than

 (verbo

(verbo venia) a mere barbarism. I do not, however, despair of seeing vacciola the *m*umpsimus, that will expel the *s*umpsimus vaccina; so strenuously is the former protected and promoted.

Dr. W. does me the honor of wishing me to be a compound maker; what thinks he then of *Bounosus* (a dactyle and perfectly euphonious) for cow-pock; *bounosize*, to (suppose) *vacciolate* or inoculate; *bounosism*, vaccinoculation; and *bounoserous*, for *vaccærarous*, matter? I abide by the decision of the College of Physicians; but, *manum de tabula;* for Dr. W. may be assured, that I am no maker of medical compounds. *Quod medicorum est promittunt medici;*—not, Your's, &c.

